# Promoting Physical Activity Through Conversational Agents: Mixed Methods Systematic Review

**DOI:** 10.2196/25486

**Published:** 2021-09-14

**Authors:** Tiffany Christina Luo, Adrian Aguilera, Courtney Rees Lyles, Caroline Astrid Figueroa

**Affiliations:** 1 School of Social Welfare University of California, Berkeley Berkeley, CA United States; 2 Department of Psychiatry Zuckerberg San Francisco General Hospital University of California, San Francisco San Francisco, CA United States; 3 Center for Vulnerable Populations Zuckerberg San Francisco General Hospital University of California, San Francisco San Francisco, CA United States; 4 Department of Epidemiology & Biostatistics University of California, San Francisco San Francisco, CA United States; 5 Department of Medicine University of California, San Francisco San Francisco, CA United States; 6 School of Public Health University of California, Berkeley Berkeley, CA United States

**Keywords:** physical activity, health behavior, behavior change, conversational agent, virtual agent, chatbot, digital health, eHealth, mHealth, mobile health, mobile phone

## Abstract

**Background:**

Regular physical activity (PA) is crucial for well-being; however, healthy habits are difficult to create and maintain. Interventions delivered via conversational agents (eg, chatbots or virtual agents) are a novel and potentially accessible way to promote PA. Thus, it is important to understand the evolving landscape of research that uses conversational agents.

**Objective:**

This mixed methods systematic review aims to summarize the usability and effectiveness of conversational agents in promoting PA, describe common theories and intervention components used, and identify areas for further development.

**Methods:**

We conducted a mixed methods systematic review. We searched seven electronic databases (PsycINFO, PubMed, Embase, CINAHL, ACM Digital Library, Scopus, and Web of Science) for quantitative, qualitative, and mixed methods studies that conveyed primary research on automated conversational agents designed to increase PA. The studies were independently screened, and their methodological quality was assessed using the Mixed Methods Appraisal Tool by 2 reviewers. Data on intervention impact and effectiveness, treatment characteristics, and challenges were extracted and analyzed using parallel-results convergent synthesis and narrative summary.

**Results:**

In total, 255 studies were identified, 7.8% (20) of which met our inclusion criteria. The methodological quality of the studies was varied. Overall, conversational agents had moderate usability and feasibility. Those that were evaluated through randomized controlled trials were found to be effective in promoting PA. Common challenges facing interventions were repetitive program content, high attrition, technical issues, and safety and privacy concerns.

**Conclusions:**

Conversational agents hold promise for PA interventions. However, there is a lack of rigorous research on long-term intervention effectiveness and patient safety. Future interventions should be based on evidence-informed theories and treatment approaches and should address users’ desires for program variety, natural language processing, delivery via mobile devices, and safety and privacy concerns.

## Introduction

### Background

Physical activity (PA) is crucial to health and well-being, and regular exercise can reduce the risk of disease, improve mental health, and boost quality of life [1]. In 2016, 28% of adults globally did not meet the World Health Organization’s PA guidelines for 150 minutes of aerobic activity per week [2]. Global PA levels have not improved since 2001, and the prevalence of inactivity has steadily risen in high-income countries [2]. Therefore, innovative interventions are required to increase PA.

Recently, there has been an increase in digital health interventions that promote healthy lifestyle changes through technologies such as smartphone apps, web-based programs, and text messages [3]. Some of these interventions are as effective as in-person interventions at modifying behavior [4]. Programs may include virtual health coaching, workout or diet plans, progress monitoring, and positive reinforcement for healthy eating and PA. Tailored feedback based on individual goals, habits, and circumstances can create a more personalized experience for users. Furthermore, some digital platforms offer users the option of pairing activity trackers such as pedometers, accelerometers, and heart rate monitors to improve the accuracy of data tracking and performance feedback.

In addition to their customizability, digital interventions allow health programs to have a wide reach. In 2018, mobile phone ownership rates ranged from 83% in emerging economies to >90% in advanced economies worldwide [5]. Smartphone ownership and internet use are nearly universal in most advanced economies and continue to grow rapidly in emerging economies [5]. With the advent of technology, demographic groups that previously did not have access to health coaching because of prohibitive costs can now access that support. Low-income Hispanic adults and Black adults in the United States, in particular, may benefit, as they have a significantly higher prevalence of physical inactivity than non-Hispanic White adults [6]. Smartphone ownership and use are more common in Hispanic and Black households than in non-Hispanic White households [7], making mobile platforms suitable for disseminating health-related interventions to underserved communities.

Digital interventions can take the form of a conversational agent, also known as a chatbot or virtual agent. Conversational agents are software programs that mimic written or spoken human conversations. They come in many forms, from chatbots engaging in written conversations to avatars simulating face-to-face discussions through synthetic speech [8]. Depending on their form, conversational agents may be deployed through standalone computer software, messaging apps, web-based platforms, mobile apps, and SMS text messaging or multimedia messaging services (MMSs). Interacting with conversational agents typically does not require much digital literacy beyond chatting or typing.

Simple conversational agents operate according to expert systems or rule-based systems, meaning they generate conversations based on questions and responses written by program developers [9]. In such cases, users are often restricted to selecting predefined answers. Conversational agents with more advanced capabilities are programmed to conduct natural language processing and integrate machine learning. Users are free to enter any command, and conversational agents formulate appropriate responses based on artificial intelligence algorithms.

Conversational agents have been increasingly used in the health care sector to help patients achieve their health goals, owing to their ability to provide interactive and personalized content [8]. Many of these conversational agents provide daily feedback, encouragement, and adaptive goals based on objective data received from fitness trackers. In contrast to in-person health coaching, conversational agents can be accessed around the clock for the duration of the intervention.

An example of a conversational agent that supports individuals in reaching their health goals is Ally, a smartphone-based chatbot that incorporates self-monitoring prompts, exercise planning, and financial incentives (cash and donations to a charity organization) to motivate users to walk more [10]. Another example, FitChat, uses goal setting, discussions of barriers, and motivational messages to encourage older adults to engage in aerobic activity and muscle-strengthening exercises [11]. A third example, Laura, falls into the subset of conversational agents termed *relational agents* [12-14]. Relational agents are computational artifacts, often with humanlike appearance and speech, designed to establish social-emotional relationships with users [12]. Relational agents such as Laura use social dialog, empathy, humor, and self-disclosure to keep users engaged over time and motivate them to create and maintain exercise habits [12].

### Rationale

Systematic and scoping reviews have been conducted on the use of digital interventions to increase PA [15-18] and the use of conversational agents in health care [8,19-21]. Previous reviews have found that many digital interventions are not theoretically based or evidence informed [4]. These interventions may be limited in their impact, as they do not include established constructs for behavior change. Although there is emerging evidence that most behavior change interventions are suitable for adaptation to a digital platform [22], few studies have addressed how digital content is linked to empirically tested frameworks and how program content and dialog flows are translated from face-to-face to virtual delivery.

It is unknown whether previous findings extend to PA conversational agents. To our knowledge, no systematic reviews have focused exclusively on PA conversational agents and analyzed their use of theories, treatment approaches, and intervention components. Research in this domain may help elucidate the successes and shortcomings of current interventions, thus guiding the development of program content and dialog flows that will have maximum impact on users.

### Objectives

Our objective is to conduct a systematic review to (1) summarize the usability and effectiveness of PA conversational agents; (2) describe common theoretical frameworks, treatment approaches, and intervention techniques; and (3) identify areas for further development.

## Methods

### Overview

We conducted a mixed methods systematic review following the Preferred Reporting Items for Systematic Reviews and Meta-Analyses guidelines [23] (Multimedia Appendix 1 [23]). The protocol for this systematic review was registered on the Open Science Framework registries [24].

We chose a mixed methods systematic review as conversational agents are still relatively new. As such, there is a shortage of randomized controlled trials (RCTs) investigating their efficacy and effectiveness in the health care sector [8]. Many studies of conversational agents include both quantitative data (eg, step counts and participant ratings on Likert scales) and qualitative data (eg, quotes from individual interviews or focus group sessions); a mixed methods design produces a more comprehensive overview of conversational agents than synthesizing quantitative or qualitative data only.

### Eligibility Criteria

The formulation of the eligibility criteria was based on the PICOS (patient problem, intervention, comparison, outcomes, and studies) framework (Textbox 1) [25].

**Inclusion and exclusion criteria using the PICOS (patient problem, intervention, comparison, outcomes, and studies) framework.**

**Inclusion criteria**

- Patient problem: studies that targeted physical activity in users
- Intervention: interventions that involved an automated conversational agent
- Comparison: another intervention type or delivery method (eg, face-to-face and app), treatment as usual, no treatment, or one group pre-post comparison
- Outcomes: reporting of intervention impact on participants or participants’ experiences with the conversational agent; some description of theoretical basis, dialog flow development, or intervention components of the program
- Study type: quantitative, qualitative, and mixed methods studies

**Exclusion criteria**

- Patient problem: studies that did not target physical activity in users
- Intervention: interventions that did not involve an automated conversational agent
- Comparison: studies without a comparison condition were not excluded, provided they still included sufficient outcome data
- Outcomes: no mention of intervention impact or participant experiences; no description of the applied intervention
- Study type: literature reviews, conference abstracts, dissertations, protocol papers, and tutorials

The inclusion criteria for this review included primary literature that involved an automated conversational agent. We focused on studies describing existing conversational agents, as opposed to studies exploring hypothetical uses of conversational agents, in an attempt to present concrete findings with external validity. We did not place any limitations on the conversational agent type, delivery platform, dialog technique, or input and output modalities. PA had to be one of the targets of the intervention. No restrictions were imposed on the target population or setting.

Studies were excluded if there was no primary research conducted or if the intervention did not use an automated conversational agent to target PA. Studies were not excluded for the lack of a comparison condition, provided they still offered outcome data on intervention impact or participant experiences and described the intervention in sufficient detail. Protocol papers and tutorials on building conversational interfaces were excluded as they did not provide any outcome data.

### Information Sources

We searched seven relevant electronic databases (PsycINFO, PubMed, Embase, CINAHL, ACM Digital Library, Scopus, and Web of Science) from their inception through July 22, 2020. We also reviewed the reference lists of relevant papers.

### Search Strategy

We based our search strategy on a preliminary scan of the literature on digital health interventions. We also consulted a librarian at the University of California, Berkeley, to generate search strings for selected databases, using Boolean operators and thesaurus terms where applicable. We combined search terms for two major topic areas: conversational agents and PA (complete search strategy available in Multimedia Appendix 2).

### Study Selection

One author conducted the initial search in each database and imported all references into Covidence (Veritas Health Innovation), a web-based software program that facilitates collaboration among reviewers. Duplicate records were identified and removed.

The titles and abstracts of all the citations were independently screened by 2 authors for eligibility. Potentially relevant articles were retrieved in full for review. Full-text studies that did not meet the predefined eligibility criteria were excluded. Any discrepancies regarding the inclusion of an article were resolved through discussion between the 2 reviewers. Cohen κ was calculated to measure intercoder agreement.

### Data Management and Collection

Data from the selected studies were charted in a spreadsheet developed by the authors for this review (Multimedia Appendix 3). Data extraction was performed by one reviewer, with a second reviewer cross-checking the data extraction table for accuracy.

### Data Items

#### Descriptive Data

The following descriptive data were extracted from each study: authors, publication year, title, study design, targeted behaviors (in addition to PA), population (eg, clinical vs nonclinical samples), geographic focus, initial and final sample size, conversational agent name, conversational agent type, delivery method, delivery platform, conversational agent output modality, user input modality, comparison conditions, control type, and outcome measures. Data were also analyzed for the variables given in the following sections.

#### Intervention Effectiveness and Impact

Evaluation measures for assessing changes in users’ activity levels or motivation to exercise as a result of the intervention included data derived from subjective measures (eg, questionnaires and self-reports) and objective measures (eg, pedometers).

#### Theory

Theories attempt to explain how and why a behavior occurs. Theoretical frameworks may guide the design and selection of the program content. In addition, the integration of theoretical content may boost the effectiveness of behavior change interventions [4]. Examples of established theories of PA promotion that have guided some of the interventions discussed in this review include behavior change theory, the habit formation model, and the health action process approach.

#### Dialog Flow Development

Dialog flows for conversational agents are often adapted from counseling techniques for a specific treatment approach, such as motivational interviewing or cognitive behavioral therapy. These approaches can help enhance motivation for behavior change and identify barriers to PA.

#### Intervention Components

Conversational agents implement specific program elements to help users overcome exercise barriers and increase their activity levels. Examples include health education, self-monitoring, goal setting, and exercise reminders.

#### Challenges and Areas for Improvement

Study limitations, ethical considerations, barriers to program development or implementation, and key areas for improving the conversational agent were noted.

### Outcomes and Prioritization

The primary outcomes for which we collected data were (1) usability and effectiveness of PA conversational agents; (2) theories, intervention components, and cognitive and behavioral constructs used to motivate individuals to engage in PA; and (3) challenges and areas for improvement. Quantitative and qualitative data were collected to assess the outcomes.

### Appraisal of Studies

The methodological quality of the included studies was assessed using the Mixed Methods Appraisal Tool (MMAT) [26]. The MMAT is a valid, reliable, and efficient tool that allows the simultaneous appraisal of qualitative, quantitative, and mixed methods studies [27]. The methods section of each included study was read by 2 reviewers independently, and each study was categorized as qualitative research, RCT, nonrandomized study, quantitative descriptive study, or mixed methods study. Then, studies were rated based on their fulfillment of the MMAT criteria in each of their respective categories. Examples of methodological quality indicators include the appropriateness of study design, choice of sampling strategy, adherence to data collection methods, intervention integrity, and integration of results. Any disagreements on ratings were resolved through discussion between the 2 reviewers.

Assigning studies an overall numerical score based on the ratings of each criterion is discouraged because a single number cannot provide insight into which aspects of the study methodology are problematic [26]. Instead, we classified studies as having lower methodological quality when they met ≤60% of the MMAT criteria and higher quality when they met >60% of the criteria. In addition, we included a detailed overview of our ratings of each criterion. All eligible studies were discussed in this review regardless of their MMAT ratings, as it is discouraged to exclude studies on the basis of low methodological quality [28].

### Data Synthesis

A meta-analysis was not conducted because of the heterogeneity of study types and outcome data. Instead, data were analyzed using parallel-results convergent synthesis, which allows qualitative and quantitative evidence to be synthesized concurrently, without data transformation [29]. Parallel-results convergent synthesis is suitable for systematic reviews that pose two or more complementary review questions [29]. Following evidence synthesis, we presented a narrative summary of our findings and made recommendations for future work.

## Results

### Search Results

**Figure 1:**
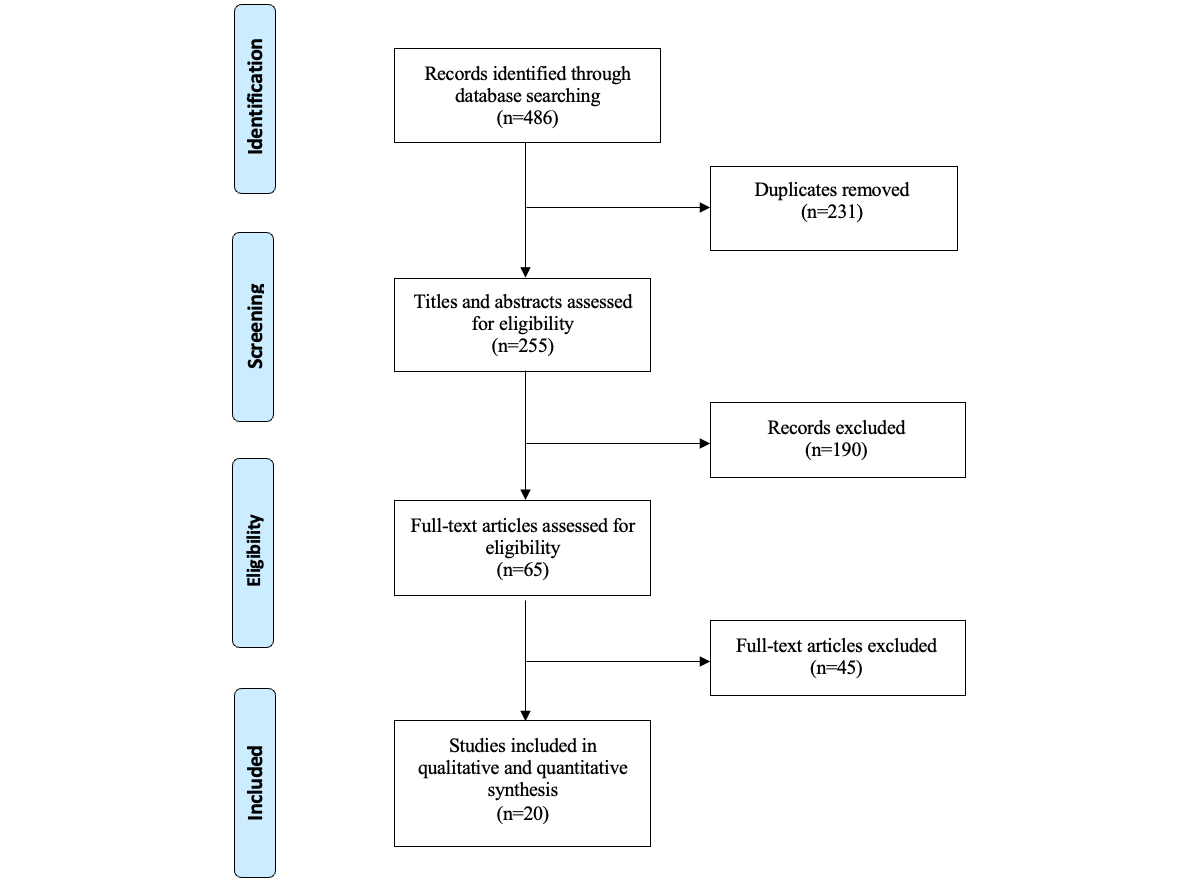
PRISMA (Preferred Reporting Items for Systematic Reviews and Meta-Analyses) flow diagram.

Our literature search retrieved 486 citations. After the removal of duplicates, 255 studies remained. An additional 74.5% (190/255) of studies were excluded after the title and abstract screening. Of the 65 remaining studies, 20 (31%) were selected for inclusion after full-text screening. Our review of the reference lists of relevant papers did not yield any additional records. The study selection process is illustrated in Figure 1. Excluded studies with reasons for exclusion are listed in Multimedia Appendix 4.

Interrater reliability was assessed at both screening stages. The *κ* coefficients were 0.71 (moderate agreement) for the title and abstract screening and 0.65 (moderate agreement) for the full-text screening.

### Overview of Included Studies

We included 20 studies evaluating 17 unique conversational agents in this review (Table 1) [12-14,30-46]. Out of the 20 studies, 10 (50%) were RCTs, 8 (40%) were quasi-experimental studies, and 2 (10%) were qualitative studies. PA was the sole target of intervention in half of the studies [12,13,32,35,37,38,41,43,45,46]. In the other half of the studies, PA was a primary target, but there were additional targets such as diet [33,34,36,39,44], fruit and vegetable consumption [30,31,40], medication adherence [14], mental well-being [33,36], stress management [33,34,36,44], and sun protection [42]. A total of 60% (12/20) studies used subjective measures to gauge intervention effectiveness and user satisfaction, and the other 40% (8/20) studies relied on objective data from pedometers or accelerometers.

The studies were conducted in 8 different countries. Studies were primarily conducted in nonclinical populations (eg, healthy adults and college students), with only 5 studies recruiting from clinical settings (eg, clinics and hospitals) [12,14,32,36,45]. The sample size ranged from 4-958 participants (median 55; mean 117, SD 206.3). Half of the studies were published in the last 3 years (2017-2020 [33,34,36-42,46]), and the other half were published between 2005 and 2014 [12-14,30-32,35,43-45].

**Table 1:** Study characteristics.

Characteristics and study			Targeted behaviors		Population		Location		Initial sample size^a^, n		Final sample size^b^, n (%)
**RCT^c^**											
	Bickmore et al [12]	PA^d^		Geriatric ambulatory clinic patients		United States		21		16 (76.2)	
	Bickmore et al [13]	PA		Healthy adults		United States		101		91 (90.1)	
	Bickmore et al [31]	PA and fruit or vegetable consumption		Healthy adults		United States		122		113 (92.6)	
	Bickmore et al [32]	PA		Geriatric ambulatory clinic patients		United States		263		250 (95.1)	
	Friederichs et al [35]	PA		Healthy adults		Netherlands		958		500 (52.2)	
	Gardiner et al [36]	PA, diet, mental well-being, and stress		Primary care clinic patients		United States		61		57 (93.4)	
	Kramer et al [38]	PA		Insurees of an insurance company		Switzerland		274		274 (100)	
	Piao et al [41]	PA		Office employees		South Korea		121		106 (87.6)	
	Vainio et al [44]	PA, diet, and stress		Healthy adults		Finland		66		38 (57.6)	
	Watson et al [45]	PA		Hospital patients		United States		70		62 (88.6)	
**Quasi-experimental**											
	Bickmore et al [14]	PA and medication		Patients with schizophrenia		United States		20		16 (80)	
	Bickmore et al [30]	PA and fruit or vegetable consumption		Healthy adults		United States		8		8 (100)	
	Fadhil and AbuRa’ed [33]	PA, diet, mental well-being, and stress		Healthy adults		Iraq		43		43 (100)	
	Fadhil et al [34]	PA, diet, and stress		University students		Italy		22		19 (86.4)	
	Kocielnik et al [37]	PA		Healthy adults		United States		33		33 (100)	
	Maher et al [39]	PA and diet		Healthy adults		Australia		31		28 (90.3)	
	Olafsson et al [40]	PA and fruit or vegetable consumption		College students		United States		39		39 (100)	
	Zhou et al [46]	PA		Chinese adults living in the United States		United States		49		49 (100)	
**Qualitative**											
	Sillice et al [42]	PA and sun protection		Healthy adults		United States		34		34 (100)	
	Simila et al [43]	PA		Older adults in exercise groups or home care		Finland		4		4 (100)	

^a^Number of participants who began the study.

^b^Number of participants who completed the intervention.

^c^RCT: randomized controlled trial.

^d^PA: physical activity.

### Results of Appraisal

Of the 20 included studies, 10 (50%) were categorized as quantitative research (RCT or nonrandomized study), 8 (40%) as mixed methods studies, and 2 (10%) as qualitative research. Overall, the methodological quality of the 20 studies varied: 55% (11/20) of the studies met ≤60% of the criteria outlined by the MMAT (lower methodological quality), and 45% (9/20) of the studies met >60% of the criteria (higher methodological quality). Reviewers’ ratings for each methodological quality criterion are presented in Multimedia Appendix 5 [12-14,30-46].

### Overview of Conversational Agents

The 20 included studies evaluated 17 unique conversational agents (Table 2). A conversational agent, Laura, was used in 15% (3/20) of the studies [12-14], and another agent, Karen, was used in 10% (2/20) of the studies [30,31]. Conversational agents Steps to Health [32], Gabby [36], Emily [40], and Elsie/Meimei [46] were designed with similar architectural systems; however, they used distinct dialog flows tailored to different populations (eg, older adults, racially diverse city-dwelling women, and Chinese adults living in the United States), so they were categorized as unique agents. For example, the conversational agent developed for racially diverse city-dwelling women delivered culturally aware patient strategies and health information and mentioned prayers and spiritual traditions [36]. Similarly, the conversational agent developed for Chinese adults emphasized values common to the Chinese culture, including collectivism [46].

**Table 2:** Conversational agent characteristics.

Conversational agent or program name		Delivery method (computer or phone)	Delivery platform	Conversational agent output (speech or text)	User input (constrained or unconstrained)
**ECA^a^**					
	Laura or FitTrack [12-14]	Computer	Software	Speech	Constrained
	Karen [30,31]	Computer	Software	Speech	Constrained
	Steps to Health [32]	Computer	Software	Speech	Constrained
	Gabby [36]	Computer	Web-based	Speech	Constrained
	Emily [40]	Computer	Software	Speech	Constrained
	Project RAISE [42]	Computer	Software	Speech	Not specified
	Virtual Coach [45]	Computer	Software	Speech	Constrained
	Elsie or Meimei [46]	Computer	Software	Speech	Constrained
**Chatbot**					
	Ollobot [33]	Both	Messaging app	Text	Unconstrained
	CoachAI [34]	Both	Messaging app	Text	Unconstrained
	Reflection Companion [37]	Phone	SMS or MMS^b^	Text	Unconstrained
	Ally [38]	Phone	Mobile app	Text	Constrained
	Paola or MedLiPal [39]	Both	Messaging app	Text	Unconstrained
	Healthy Lifestyle Coaching Chatbot [41]	Both	Messaging app	Text	Unconstrained
**Both**					
	I Move [35]	Computer	Web-based	Text	Both
	AmIE Project [43]	Computer	Software	Both	Constrained
	Mindless Change [44]	Phone	Mobile app	Both	Constrained

^a^ECA: embodied conversational agent.

^b^MMS: multimedia messaging service.

Of the 17 conversational agents, 10 (59%) were computer-based [12-14,30-32,35,36,40,42,43,45,46], 4 (24%) could be used on computers or phones [33,34,39,41], and 3 (18%) were designed for mobile devices only [37,38,44]. Conversational agents were implemented using standalone computer software [12-14,30-32,40,42,43,45,46], messaging apps [33,34,39,41], web-based platforms [35,36], mobile apps [38,44], and SMS text messaging or MMS [37].

In total, of the 17 agents, 8 (47%) were embodied conversational agents (ECAs) with synthesized speech [12-14,30-32, 36,40,42,45,46], 6 (35%) were text-only chatbots [33,34,37-39,41], and 3 (18%) had both an ECA and chatbot option [35,43,44]. With all 17 conversational agents, participants gave input by typing on a keyboard or selecting answer options with a mouse, touchpad, or touchscreen; 59% (10/17) of the conversational agents limited users to constrained input, whereby users selected answers from a multiple-choice list of options, and conversational agents responded according to predefined templates [12-14,30-32,36,38,40,43-46]. Only 29% (5/17) of the conversational agents accepted free-text responses and used machine learning and natural language processing to understand users’ input and generate replies [33,34,37,39,41], and 6% (1/17) of the conversational agents accepted free-text responses and multiple-choice answers [35]. The remaining 6% (1/17) conversational agents did not specify what user inputs were accepted [42].

### Intervention Effectiveness and Impact

#### RCTs

Of the 10 RCTs, 6 (60%) found that participants in the conversational agent group outperformed participants in the control group on various PA measures. Intervention groups increased daily walking more quickly [31], achieved >30 minutes of exercise or 10,000 steps per day more times per week [13], significantly increased step count during the study period [12,32], significantly increased self-reported PA at 1 month [35], and maintained step counts throughout time [45]. Only 10% (1/10) RCTs did not find significant differences in activity levels between the intervention and control groups [36].

The remaining 30% (3/10) of RCTs used conversational agents in both experimental and control groups but varied the conversational agent conditions (eg, cash incentives vs charity incentives vs no incentives [38], rewards vs no rewards [41], and ECA vs text-only chatbot [44]). In 67% (2/3) of these studies, interacting with a conversational agent significantly increased step counts and self-reported activity across all conditions; however, including financial incentives and rewards further boosted activity levels [38,41]. The last RCT determined that conversational agents were useful but limited by low adherence [44].

#### Quasi-Experimental Studies

Of the 8 quasi-experimental studies, 6 (75%) used within-subjects pre-post designs [14,30,33,34,37,39] and 2 (25%) included comparator groups [40,46] (Multimedia Appendix 6 [12-14,30-46]). Of the 8 quasi-experimental studies, 3 (38%) measured changes in activity level as a result of interacting with a conversational agent [14,34,39]; 2 found positive impacts in the form of increased enjoyment during walking [14], higher frequency of step-goal achievement [14], and increased weekly exercise time [39], and 1 did not find any differences in activity levels [34].

An additional 38% (3/8) of the quasi-experimental studies measured participants’ attitudes toward exercise before and after the intervention [37,40,46]. Conversational agents successfully triggered reflection on new exercise routines [37], increased participants’ self-efficacy and motivation to exercise for at least 30 minutes every day [40] and persuaded participants to start regular exercise [46].

The remaining 25% (2/8) of the quasi-experimental studies discussed users’ preliminary experiences with conversational agents [30,33]. Overall, these conversational agents had moderately high usability and feasibility. Participants perceived them to be satisfactory [30,33], trustworthy [30], empathetic [30], useful [33], and easy to use [33].

#### Qualitative Studies

Of the 20 included studies, only 2 (10%) were qualitative studies [42,43]. In one study, most participants had positive, satisfying interactions with the relational agent and found the agent humanlike, caring, and supportive [42]. About half of the participants viewed the relational agent as informative and felt motivated to maintain regular exercise. Another qualitative study compared two different PA conversational agents: a text-based chatbot and an ECA [43]. Participants had positive experiences with both systems and felt that conversational agents could provide motivation and serve as information channels.

#### ECAs Versus Chatbots

ECAs and text-only chatbots performed similarly, with 88% (7/8) of the ECAs and 83% (5/6) of the chatbots positively affecting participants’ PA levels, motivation to exercise, or perceptions of conversational agents. Of all 20 studies, 3 (15%) directly compared ECAs with chatbots; one study found that both were equally effective at building social relationships and increasing PA [35], one study suggested that ECAs could provide a slightly more engaging user experience than chatbots [42], and the remaining study described the benefits and drawbacks of each conversational agent [43].

### Intervention Characteristics

#### Theory

Of the 20 studies, 11 (55%) cited a theory that guided their intervention development (Table 3). Of these 11 studies, 6 (55%) designed the intervention and selected program elements according to the referenced theories [37,38,40,41,44,46], and 5 (45%) mentioned a theory as their overarching framework but did not explicitly link intervention components with corresponding theoretical constructs [13,30,31,34,42].

The used theories could be broadly categorized into learning theories, which describe how people receive and process knowledge, and behavior change theories, which explain how behaviors develop and shift throughout time. Four interventions were based on a combination of theories [30,31,40,44], and 1 intervention used the Hofstede cultural dimensions theory to develop culturally appropriate dialog for an American and a Chinese conversational agent [46].

**Table 3:** Distribution of theories.

Theoretical model or framework		Study
**Learning theories**		
	Learning theory (broad) [47]	Kocielnick et al [37]
	Social learning theory [48]	Bickmore et al [13]
	Social cognitive theory [49]	Bickmore et al [30,31]
	Constructivist learning theory [47]	Vainio et al [44]
	Cognitive dissonance theory [50]	Olafsson et al [40]
**Behavior change theories**		
	Behavior change theory (broad) [51]	Bickmore et al [31], Kramer et al [38]
	Habit formation model [52]	Piao et al [41], Vainio et al [44]
	Health action process approach [53]	Fadhil et al [34]
	Transtheoretical model [54]	Bickmore et al [30,31], Olafsson et al [40], Sillice et al [42]
**Other**		
	Hofstede’s cultural dimensions theory [55]	Zhou et al [46]

#### Dialog Flow Development

Of the 20 studies, 9 (45%) discussed the use of one or more treatment approaches to guide the development of dialog flows for conversational agents. The most commonly used approach was motivational interviewing [30,31,35-37,40], followed by cognitive behavioral therapy [13,33,34,45] and behavioral therapy [13,45].

Of the 9 studies, 4 (44%) described how dialog flows were adapted from face-to-face counseling and prepared for virtual delivery. Techniques included using transcripts from videotaped counseling sessions as a basis for the conversational structure [30,40], using a dialog interpreter to convert statements from counseling sessions into interactive virtual conversations [31], and developing scripts through literature reviews and consultations with physicians, computer scientists, and exercise trainers [45]. The remaining 56% (5/9) studies did not explain how dialog flows for conversational agents were written.

#### Intervention Components

The most common program components were health education, motivational messages, problem-solving barriers to exercise, goal setting, self-monitoring, and exercise tips (Table 4). Additional components included reminders, homework, workout planning, incentives, and reflection.

Participants found health education helpful [36,42], as it allowed them to learn new ways of increasing PA [40]. They also enjoyed receiving tips for new exercise routines [40] and periodic exercise reminders [31,42]. Positive feedback motivated participants [37], built rapport [42], and increased agent likeability [31]. Participants appreciated progress tracking features [34] and visual step charts [31,32]. Conversational agents helped participants formulate concrete goals, action plans, and overcome obstacles [37]. However, participants mentioned that they would have liked to talk more about how their health problems affected their ability to exercise [12]. Change talk and reflection helped participants increase their commitment to positive health behaviors [37,40]. Finally, rewards were implemented with moderate success, with one study finding that daily cash incentives increased step-goal achievement by 8.1% [38] and another study finding that intrinsic rewards improved habit formation and enhanced intervention sustainability [41].

**Table 4:** Distribution of intervention components.

Study	Goal setting	Positive reinforcement	Self-monitoring	Problem-solving barriers	Education	Tips	Reminders	Homework	Workout planning	Rewards	Change talk or reflection (motivational interviewing)
Bickmore et al [12]	✓^a^	✓	✓	✓	✓	✓					
Bickmore et al [13]	✓	✓	✓	✓	✓						
Bickmore et al [14]	✓	✓	✓	✓	✓	✓					
Bickmore et al [30]	✓	✓		✓	✓	✓		✓			✓
Bickmore et al [31]	✓			✓	✓			✓			
Bickmore et al [32]	✓	✓	✓	✓		✓					
Fadhil and AbuRa’ed [33]		✓	✓		✓	✓			✓		✓
Fadhil et al [34]		✓	✓		✓	✓	✓				
Friederichs et al [35]		✓		✓	✓				✓		✓
Gardiner et al [36]	✓		✓	✓	✓	✓		✓			
Kocielnick et al [37]	✓		✓	✓							✓
Kramer et al [38]	✓		✓	✓		✓			✓	✓	
Maher et al [39]	✓		✓	✓	✓						
Olafsson et al [40]		✓			✓						✓
Piao et al [41]	✓	✓					✓			✓	
Sillice et al [42]		✓	✓	✓			✓	✓			
Simila et al [43]		✓	✓		✓	✓	✓				
Vainio et al [44]	✓	✓	✓		✓	✓			✓		✓
Watson et al [45]	✓	✓	✓	✓	✓	✓					
Zhou et al [46]				✓	✓				✓		

^a^Intervention component present.

### Challenges and Areas for Improvement

#### Conversational Agent Constraints

The most common challenges were related to the capabilities of conversational agents. In 59% (10/17) of the conversational agents, users were required to respond via multiple-choice answers. This format limited user freedom [12,13] and lacked the personalization necessary to address more complex issues [14]. Although researchers acknowledged the need for more sophisticated dialog systems, they were concerned about the difficulty of implementing machine learning and the increased chance of misunderstanding users’ intents [13].

Another area for improvement was communication modality. None of the conversational agents were built to accept spoken input. Participants were required to type out their answers or select answers using a mouse, touchpad, or touchscreen. In one study, participants universally stated that they would have preferred speaking to the conversational agent [12].

Studies have presented mixed findings on the value of ECAs with synthesized speech. According to qualitative data, talking ECAs seemed more versatile than text-only chatbots [43] and provided a closer approximation of face-to-face conversations with health care providers [32]. However, 45% (5/11) ECAs were criticized by participants for their robotic voices, slow pace, unnatural movements, and limited relational skills [35,36,40,42,46].

#### Program Delivery

Participants encountered more issues with computer-based than with phone-based conversational agents. Some participants had limited access to computers, limited time to sit in front of computers [36], or difficulties installing software and entering information [12]. Internet access was also an issue, with network breaks preventing participants from starting apps, synchronizing devices and databases, and connecting fitness trackers [43]. Many participants across studies felt that having the conversational agent on their phone would be more convenient and accessible, allowing them to complete the program “on the go” [32,36,42].

Mobile interventions were well-liked, particularly those that used familiar messaging apps, as they did not require participants to download and learn to use additional applications [41]. However, some participants had minimal smartphone skills and did not know how to send text messages, thus limiting their engagement with the intervention [39]. In addition, one mobile app suffered from poor usability because of slow performance on older smartphones [44].

#### Program Content

Of the 20 studies, 7 (35%) studies mentioned the repetitiveness of program content as a key area for improvement [12,13,30,31,37,42,43]. This included dialog flows that were often repeated, leading to lower satisfaction [30] and increased boredom [37,43]. Participants desired more personalized responses and suggestions based on their health information, preferences, and PA history [37,40]. Owing to repetitiveness, participants felt that continued use would not lead to any additional impact [42].

User engagement waned throughout time [45], and high attrition rates limited the efficacy of the interventions. In one study, participants responded to 50% of the self-monitoring prompts and completed only a few exercise and coping plans, explaining that weekly planning was too difficult and time-consuming [38]. In another study, participants found the conversational agent engaging, but without external support, almost half of them discontinued the use of the service [44]. Participants who lapsed for a short period were more likely to quit the program [41].

#### Ethical Issues

Many relational agents relied on social dialog, humor, empathic statements, and personal stories to build rapport with users [14,31,32,42,46]. The use of these techniques may have increased the potential for misperceptions and false illusions, as virtual agents do not have emotions or personal histories. Humans tend to anthropomorphize advanced technology [13], and conversational agents may have deceived some users into thinking they were interacting with a human. One study pointed out that patients with schizophrenia who are experiencing a psychotic episode could be more likely to confuse relational agents with real people, develop parasocial relationships with relational agents, or become paranoid that relational agents or their programmers are monitoring their behavior [14]. Researchers attempted to address this matter by having the relational agent periodically remind users that it was “just a computer character with limited capabilities” [14].

#### Standards of Care

Of the 20 studies, only 1 (5%) compared the quality of care between a human and a conversational agent. This study found that a human agent was often more motivating, engaging, and supportive than a virtual agent [34].

Most studies did not address privacy features or data storage and access procedures despite participants expressing concerns that conversational agents could collect and share their personal information [12,14]. One study discussed security measures, such as requiring usernames and passwords and automatically logging users out after a period of inactivity [36]. Another study described weekly backup procedures to mitigate the possibility of data loss due to system crashes or computer theft [12].

Finally, 10% (2/20) of studies discussed user safety issues. One conversational agent provided videos demonstrating exercises that a participant with arthritis could not safely perform without the help of an elastic band [43]. Another study discussed the necessity of improving automated dialog flows because of conversational agents’ inadequate responses to safety concerns mentioned in users’ free-text answers [40].

## Discussion

### Principal Findings

This literature review charted data from 20 studies that evaluated 17 PA conversational agents. Overall, conversational agent interventions were feasible and promising for increasing PA. Of the 10 RCTs, 6 (60%) found that participants assigned to the conversational agent group outperformed participants in the control group on PA measures, such as step counts and exercise frequency and duration. Conversational agents had moderate usability and acceptability, as measured by subjective data in the form of questionnaires, interviews, activity logs, and diaries. The interventions were generally found to be useful, easy to use, and satisfactory to participants; however, they faced some implementation challenges, including high attrition, technical issues, limited options for user input, and privacy and security risks. Methodological quality varied across studies, and few studies adequately addressed issues of user engagement, safety, and ethics.

### Comparison With Prior Work

To the best of our knowledge, this is the first systematic review to evaluate PA conversational agents. Previous reviews have reported on the effectiveness of digital interventions for increasing PA [15-18]. Our results are consistent with their findings that digital interventions have a modest effect on activity levels, particularly in the short term; however, user engagement tends to decline over time [16-18]. Our findings are also in line with other reviews’ evaluations of health care conversational agents, which show that natural language processing and machine learning are underused, high-quality evidence and attention to patient safety are lacking, and study methods and evaluation measures are often inconsistently reported [8,20,21].

### Recommendations

On the basis of the findings of this review, we propose several recommendations for the future design and implementation of PA conversational agents.

#### Program Content

Participant feedback indicated that many intervention programs lost their novelty over time, resulting in decreased user engagement. More diverse program content is required to maintain long-term user satisfaction. A way to reduce repetitiveness is through just-in-time adaptive interventions (JITAIs), which provide dynamically tailored support when users need it while minimizing user burden [10]. JITAIs can inform participants when they have been sedentary for long periods or when they are behind on their step goals. In addition, JITAIs can offer exercise suggestions based on weather conditions, time of day, and users’ physical surroundings. JITAIs for conversational agents are currently being explored and developed through microrandomized trials [10].

Another way to improve the sustainability of interventions is to base their programming on relevant behavior change theories and evidence-based treatment approaches. Behavior change theories may help identify intervention techniques that tap into users’ motivations and result in increased engagement. Similarly, dialog flows based on treatment approaches, such as motivational interviewing and cognitive behavioral therapy, can help users explore and resolve barriers to PA. Owing to the heterogeneity of the studies, we were unable to determine if the inclusion of a theoretical framework or treatment approach increased intervention effectiveness in this review. Future work should assess this as the number of studies increases.

Programming conversational agents to send periodic tips and exercise reminders may help decrease the high attrition rates reported in a few studies [38,41,44,45]. In addition, as many PA interventions are self-guided, encouraging users to share goals and progress with their social circles may increase accountability.

#### Conversational Agent Delivery

Computer-based ECAs were the most common agents used; however, qualitative interviews revealed that participants desired mobile delivery platforms. Phone ownership rates are higher than computer ownership rates [7]; thus, conversational agents operating via SMS or MMS text messaging may increase scalability. They are also appropriate for those with low digital literacy. For computer-based agents, web-based platforms and familiar messaging apps that do not need to be installed or regularly updated may be more accessible than standalone software.

ECAs have the potential to improve human-computer interactions; however, they are commonly criticized as robotic and unnatural. ECAs can be improved by replacing synthesized speech with human voice, giving users control over pacing of messages, and designing higher-quality animation. Automatic speech recognition is highly desirable, particularly among populations with low vision or difficulty typing. In addition, although artificially intelligent conversational agents may take more time to develop, they afford users more freedom and personalized content to sustain engagement and maximize treatment efficacy.

#### Safety and Ethics

Most conversational agent programs were designed for healthy and able-bodied adults; however, programs should also be equipped with education and exercise tips for users of different age groups and users with physical limitations. Conversational agents should offer suggestions for exercise-related injuries or pain, such as performing pre- and postworkout stretches, modifying activities, and consulting with health care providers. Users may mention mental health conditions such as depression or anxiety that prevent them from exercising. Thus, researchers should consider incorporating dialog flows that refer users to mental health resources and crisis hotlines. If interventions are designed specifically for clinical populations, additional safety features may be necessary, such as periodic check-ins with a human advisor. Furthermore, for individuals with severe mental illnesses, such as psychosis, additional consideration may be warranted, including ensuring agents are not too anthropomorphic.

Users often share sensitive health information with conversational agents. However, only a few studies have discussed privacy and security issues. User privacy should be protected through measures such as requiring logins and passwords for apps and software, deidentifying user data, and archiving past conversations.

Finally, efforts must be made to uphold the quality of digital interventions. There are currently no regulations regarding the standards of care for conversational agents. Similar to health interventions provided by human coaches, conversational agent programs should be based on a relevant theory and treatment approach to ensure that they are grounded in evidence-based practice.

### Limitations

The findings of this review must be considered in the context of a few limitations. First, we may have missed relevant studies in additional databases despite our search strategy being fairly broad. In particular, we lacked quantitative descriptive studies and qualitative studies without comparison conditions, which could suggest that our PICOS criteria were better suited for effectiveness studies that included comparison conditions. Although we aimed to include usability studies without comparison conditions, we had to exclude many such studies because of insufficient data on study participants’ experiences, the intervention’s impact on activity levels, or the intervention’s theoretical mechanisms of change.

Second, because of the heterogeneity of study designs and outcome data, we could not conduct a meta-analysis or directly compare different interventions. We synthesized the main findings from the existing literature; however, without effect sizes, it was difficult to draw definitive conclusions about intervention effectiveness. This field of research would benefit from more longitudinal RCTs that evaluate the long-term sustainability of conversational agents.

Third, we appraised the methodological quality of the included studies following the appropriate method-related standards using the MMAT, one of the few tools designed specifically for mixed methods reviews. However, the MMAT is not designed to grade the level of evidence or the risk of bias in effectiveness studies. We chose not to apply an ad hoc tool to appraise the risk of bias of effectiveness studies because only half of the included studies were RCTs that reported on treatment effectiveness. To date, there is no single, unified approach for assessing confidence in findings generated from combined quantitative and qualitative evidence [56]. More research is needed on best practices for critically appraising included studies in mixed methods reviews.

Fourth, we refrained from analyzing the more technical aspects of conversational agents (eg, programming and interfaces), choosing instead to focus on intervention components and guiding frameworks. Additional questions regarding technical design should be studied in systematic reviews to maximize the user-friendliness of conversational agents.

Fifth, intervention techniques were difficult to identify, as some studies embedded them within figures rather than discussing them descriptively, and there was no uniform language across studies regarding techniques.

Finally, more than half of the included studies focused exclusively on healthy adults, thus limiting the generalizability of their results. As conversational agents are often designed for a broad audience, future studies should also consider sampling from youth and clinical populations (eg, individuals with mental illness or pre-existing health conditions).

### Conclusions

On the basis of current evidence, conversational agents appear to be a feasible and effective modality for delivering PA interventions. However, more research comparing conversational agents with other forms of interventions, including human-delivered interventions, is required. Most conversational agents reviewed were computer-based and constrained users to written, predefined inputs. Future conversational agents should consider accessibility and inclusive design and consider supporting automatic speech recognition, natural language processing, and mobile phone platforms. In addition, program content should be further personalized and diversified by using relevant evidence-based frameworks and their accompanying behavior change methods. Researchers should provide a clear overview of how they select intervention components and how these components affect health behavior. This can lead to a deeper understanding of the mechanisms of change in interventions, and consequently, increase the effectiveness of these interventions. Personalization of program content may also lead to higher user satisfaction and engagement while supporting user choice and agency. Finally, in addition to user experiences, safety, privacy, and ethical concerns should be prioritized in the design of PA conversational agents.

## Appendix

Multimedia Appendix 1 PRISMA (Preferred Reporting Items for Systematic Reviews and Meta-Analyses) checklist.

Multimedia Appendix 2 Search strategy.

Multimedia Appendix 3 Data extraction form with descriptions of data items.

Multimedia Appendix 4 Excluded studies with reasons for exclusion.

Multimedia Appendix 5 Mixed Methods Appraisal Tool (MMAT) quality appraisal profile.

Multimedia Appendix 6 Characteristics of comparators in each included study.

## Abbreviations

ECA: embodied conversational agent
JITAI: just-in-time adaptive intervention
MMAT: Mixed Methods Appraisal Tool
MMS: multimedia messaging service
PA: physical activity
PICOS: patient problem, intervention, comparison, outcomes, and studies
RCT: randomized controlled trial
