# Supplementary material for: Promoting Physical Activity Through Conversational Agents: Mixed Methods Systematic Review
Source: J Med Internet Res. 2021 Sep 14;23(9):e25486. doi: 10.2196/25486 (PMC8479596; doi:10.2196/25486)
Supplement: Multimedia Appendix 1 [file jmir_v23i9e25486_app1.pdf]

Multimedia Appendix 1. PRISMA checklist.

Adapted PRISMA for reporting systematic reviews of qualitative and quantitative evidence

Section/topic	#	Checklist item	Reported on page #
**TITLE**			
Title	1	Propose a short take-home title. The title should explicitly state that the review included different type of evidence.	Title: p. 1
**ABSTRACT**			
Structured summary	2	Provide a structured summary including, as applicable: background; objectives; data sources; study eligibility criteria, participants, and interventions; study appraisal and synthesis methods; results; limitations; conclusions and implications of key findings; systematic review registration number.	Abstract: p. 1
**INTRODUCTION AND OBJECTIVES**			
Rationale	3	Describe the rationale for the review (e.g., a health problem) in the context of what is already known (e.g., an existing literature review paper or a reference book chapter).	Rationale: p. 2
Objectives	4	Formulate questions and/or objectives (qualitative, quantitative or both) being addressed by your review.	Objectives: p. 2-3
**METHODS**			
Protocol and registration	5	Indicate if a review protocol exists, if and where it can be accessed (e.g., Web address), and, if available, provide registration information including registration number.	Overview: p. 3
Justification	6	Justify the use of a review of qualitative and quantitative evidence	Overview: p. 3
Eligibility criteria	7	Specify the inclusion and exclusion criteria and the rationale for supporting these criteria.	Eligibility Criteria: p. 3
Information sources	8	Describe all information sources (e.g., databases with dates of coverage, contact with study authors to identify additional studies) in the search and date last searched.	Information Sources: p. 3
Search	9	Present full electronic search strategy for at least one database (e.g., in an appendix), including any limits used, such that it could be repeated. Describe the process for removing duplicates. Specify the involvement of a librarian, if applicable.	Search Strategy: p. 3<br>Multimedia Appendix 2
Study selection	10	Describe the process for selecting studies (e.g., screening based on titles and abstracts, and eligibility based on full-text, number of reviewers, software used).	Study Selection: p. 3-4
Data collection process	11	Describe the method of data extraction from included studies (e.g., number of reviewers involved, piloted forms, etc.). List the data extracted. If applicable, state any	Data Management and Collection: p. 4

Multimedia Appendix 1. PRISMA checklist.

Adapted PRISMA for reporting systematic reviews of qualitative and quantitative evidence

		processes for obtaining and confirming data from investigators of included studies (e.g., initial email to the first author and reminder email).	
Appraisal	12	Describe the process for appraising included studies (e.g., tools used, number of reviewers involved), and specifically for assessing the methodological quality or risk of bias of included qualitative, quantitative, and mixed methods studies. Specify how results of this appraisal are used in the synthesis. For example, for descriptive purpose (include all studies with description of their methodological quality or risk of bias) or for analytical purpose (contrast synthesis of 'lower quality' studies vs. 'higher quality' studies using sensitivity analysis).	Appraisal of Studies: p. 4
Synthesis	13	Describe the synthesis design used. Describe and justify the synthesis method(s) used (e.g., quantitative content analysis, meta-analysis, thematic synthesis, etc.)	Data Synthesis: p. 4
Additional analysis	14	Describe methods of additional analyses (e.g., sensitivity or subgroup analyses), if done.	N/A
**RESULTS**			
Study selection	15	Give numbers of studies screened, assessed for eligibility, and included in the review, with reasons for exclusions at each stage. Summarize in a flow diagram (see Appendices). Give numbers of quantitative, qualitative and mixed methods studies included.	Search Results: p. 4-5<br>Multimedia Appendix 4
Study characteristics	16	For each study, present characteristics for which data were extracted (e.g., tables of characteristics of included studies – see Appendices) and provide the citations. Specify common information across all included studies. Describe the studies including their heterogeneity (variability associated with differences between studies)	Overview of Included Studies: p. 5-6
Result of appraisal	17	Present data on the methodological quality or risk of bias of included studies based on the appraisal done.	Results of Appraisal: p. 6<br>Multimedia Appendix 5
Results of synthesis	18	Present results of synthesis. If qualitative synthesis: - In the text, briefly summarize the main themes or categories and refer to the appendix. - Appendix (table, figure, or matrix): For each study, present the themes or categories identified. If quantitative synthesis: - In the text, briefly summarize the data and refer to the appendix. - Appendix (table, figure, or matrix): For all key variables, present, for each study: (a) simple summary data for each intervention group and (b) effect estimates and confidence intervals, ideally with a forest plot. If qualitative and quantitative syntheses: - Present both - If applicable, present the results of the integration of both syntheses.	Intervention Effectiveness and Impact; Intervention Characteristics; Challenges and Areas for Improvement: p. 7-11
Additional analysis	19	Give results of additional analyses, if done (e.g., sensitivity or subgroup analyses).	N/A

Multimedia Appendix 1. PRISMA checklist.

Adapted PRISMA for reporting systematic reviews of qualitative and quantitative evidence

**DISCUSSION**			
Summary of evidence	20	Provide an overall summary of results (take-home messages) from the qualitative and/or quantitative synthesis. State the main results for each main theme or category, and/or key process/outcome variable. Consider their relevance and importance for knowledge users (e.g., health care providers, managers, and decision/policy makers). Take into account the methodological quality across studies (when applicable). Describe insight gained from the integration of qualitative and quantitative evidence.	Principal Findings: p. 11
Contribution	21	Describe the contribution of the review (compared to what is already known) with respect to: Review methods; Scientific knowledge; Practice, program planning and evaluation, policy making.	Comparison with Prior Work; Recommendations: p. 11-12
Limitations	22	Specify any element that may affect the cumulative evidence. Discuss limitations at the study and process/outcome levels (e.g., lack of rich data for qualitative synthesis, methodological quality/risk of bias, and their potential consequences on the results). Discuss limitations at the review level (e.g., dependent reviewers, incomplete retrieval of relevant studies - selective publication of reports regarding studies with positive results), and limited reporting (selective reporting of information about included studies)), and their potential consequences on the results.	Limitations: p. 12-13
Conclusions	23	Provide a general interpretation of the results in the context of other evidence, including implications for knowledge users (e.g., major recommendation). State implications for future research.	Conclusions: p. 13
**ACKNOWLEDGEMENTS**			
Acknowledgements	24	Describe sources of funding and other support (e.g., supply of data) and the role of funders in the review. Acknowledge any information about potential conflict of interest.	Acknowledgements: p. 13<br>Conflicts of Interest: p. 13
**REFERENCES**			
References	24	List all the references cited in the text.	References: p. 14-16

Adapted from: Moher D, Liberati A, Tetzlaff J, Altman DG, The PRISMA Group (2009). Preferred Reporting Items for Systematic Reviews and Meta-Analyses: The PRISMA Statement. PLoS Med 6(6): e1000097. doi:10.1371/journal.pmed1000
