# Supplementary material for: Promoting Physical Activity Through Conversational Agents: Mixed Methods Systematic Review
Source: J Med Internet Res. 2021 Sep 14;23(9):e25486. doi: 10.2196/25486 (PMC8479596; doi:10.2196/25486)
Supplement: Multimedia Appendix 2 [file jmir_v23i9e25486_app2.pdf]

Multimedia Appendix 2. Search strategy.

Database	Limits	Date conducted	Search string
PsycINFO	Peer-reviewed	Conducted July 1, 2020; updated July 22, 2020	("physical activity" OR "physical activities" OR exercise OR exercises OR exercising OR (MAINSUBJECT.EXACT("Activity Level") OR MAINSUBJECT.EXACT("Active Living") OR MAINSUBJECT.EXACT("Physical Fitness"))) AND ("conversational agent" OR "conversational agents" OR "conversational bot" OR "conversational bots" OR "dialogue system" OR "dialogue systems" OR “dialog system” OR “dialog systems” OR "chat bot" OR "chat bots" OR chatbot OR chatbots OR "relational agent" OR "relational agents" OR "virtual agent" OR "virtual agents" OR "virtual coach" OR "virtual coaches" OR "virtual coaching" OR "virtual assistant" OR "virtual assistants")
PubMed	none	Conducted July 1, 2020; updated July 22, 2020	("conversational agent" OR "conversational agents" OR "conversational bot" OR "conversational bots" OR "dialogue system" OR "dialogue systems" OR "dialog system" OR "dialog systems" OR "chat bot" OR "chat bots" OR chatbot OR chatbots OR "relational agent" OR "relational agents" OR "virtual agent" OR "virtual agents" OR "virtual coach" OR "virtual coaches" OR "virtual coaching" OR "virtual assistant" OR "virtual assistants") AND ("physical activity" OR "physical activities" OR "physical fitness" OR "activity level" OR exercise OR exercises OR exercising)
Embase	Article (under “Publication types”)	Conducted July 1, 2020; updated July 22, 2020	("conversational agent" OR "conversational agents" OR "conversational bot" OR "conversational bots" OR "dialogue system" OR "dialogue systems" OR "dialog system" OR "dialog systems" OR "chat bot" OR "chat bots" OR chatbot OR chatbots OR "relational agent" OR "relational agents" OR "virtual agent" OR "virtual agents" OR "virtual coach" OR "virtual coaches" OR "virtual coaching" OR "virtual assistant" OR "virtual assistants") AND ("physical activity" OR "physical activities" OR "physical fitness" OR "activity level" OR exercise OR exercises OR exercising)

CINAHL Complete	none	Conducted July 1, 2020; updated July 22, 2020	("conversational agent" OR "conversational agents" OR "conversational bot" OR "conversational bots" OR "dialogue system" OR "dialogue systems" OR "dialog system" OR "dialog systems" OR "chat bot" OR "chat bots" OR chatbot OR chatbots OR "relational agent" OR "relational agents" OR "virtual agent" OR "virtual agents" OR "virtual coach" OR "virtual coaches" OR "virtual coaching" OR "virtual assistant" OR "virtual assistants") AND ("physical activity" OR "physical activities" OR "physical fitness" OR "activity level" OR exercise OR exercises OR exercising)
ACM Digital Library	none	Conducted July 1, 2020; updated July 22, 2020	("conversational agent" OR "conversational agents" OR "conversational bot" OR "conversational bots" OR "dialogue system" OR "dialogue systems" OR "dialog system" OR "dialog systems" OR "chat bot" OR "chat bots" OR chatbot OR chatbots OR "relational agent" OR "relational agents" OR "virtual agent" OR "virtual agents" OR "virtual coach" OR "virtual coaches" OR "virtual coaching" OR "virtual assistant" OR "virtual assistants") AND ("physical activity" OR "physical activities" OR "physical fitness")
Scopus	none	Conducted July 22, 2020	("conversational agent" OR "conversational bot" OR "dialogue system" OR "dialog system" OR "chat bot" OR chatbot OR "virtual agent" OR "virtual coach") AND ("physical activity" OR "physical fitness" OR "active living" OR "activity level")
Web of Science	Article (under “Document Types”)	Conducted July 22, 2020	("conversational agent" OR "conversational agents" OR "conversational bot" OR "conversational bots" OR "dialogue system" OR "dialogue systems" OR "dialog system" OR "dialog systems" OR "chat bot" OR "chat bots" OR chatbot OR chatbots OR "relational agent" OR "relational agents" OR "virtual agent" OR "virtual agents" OR "virtual coach" OR "virtual coaches" OR "virtual coaching" OR "virtual assistant" OR "virtual assistants") AND ("physical activity" OR "physical activities" OR "physical fitness" OR "activity level" OR exercise OR exercises OR exercising)
