# Supplementary material for: Promoting Physical Activity Through Conversational Agents: Mixed Methods Systematic Review
Source: J Med Internet Res. 2021 Sep 14;23(9):e25486. doi: 10.2196/25486 (PMC8479596; doi:10.2196/25486)
Supplement: Multimedia Appendix 3 [file jmir_v23i9e25486_app3.pdf]

Multimedia Appendix 3. Data extraction form with descriptions of data items.

	Concept	Definition
**Study Characteristics**		
	Author(s)	Study authors.
	Publication year	The year that the study was published.
	Title	The title of the study.
	Study design	The research method that the study used to collect data (e.g., RCT, quasi-experimental study, qualitative study).
	Targeted behavior(s)	The behaviors that the intervention aimed to affect (e.g., physical activity, diet, mental wellbeing, stress).
	Population	The health status of study participants (e.g., clinical or non-clinical).
	Geographic focus/ location	The country where the researchers recruited participants and/or conducted the study.
	Initial sample size	The number of people who began the study.
	Final sample size	The number of people who completed the study.
**Conversational Agent Characteristics**		
	Conversational agent name	Name of the conversational agent and/or program.
	Conversational agent type	How the conversational agent appears on screen to users (e.g., embodied conversational agent or text-based chatbot).
	Delivery method	The technological medium through which the conversational agent can be accessed (e.g., computer or phone).
	Delivery platform	The platform by which the conversational agent is implemented (e.g., standalone computer software, web-based, mobile applications, SMS/MMS text messages).
	Conversational agent output modality	How the conversational agent interacts with the user: spoken language (via speakers) or written language (via text on the screen).
	User input modality	How the user interacts with the chatbot: constrained (predefined answer options) or unconstrained (free-text responses)
**Comparator Characteristics**		
	Comparison conditions	The number of comparison groups and the type of intervention they receive (e.g., ECA or chatbot, rewards or no rewards).

	Control type	The intervention that the control group receives (e.g., pedometer, patient education sheets, website, treatment as usual, no intervention).
**Outcomes**		
	Outcome measures	The type of data used to measure outcomes: objective data (e.g., pedometer, accelerometer) or subjective data (e.g., self-report, questionnaires, activity logs).
	Intervention effectiveness and impact	Effectiveness and impact of the intervention (e.g., increased step count, more minutes of physical activity per week, more steps walked, increased motivation to exercise).
	Theory	A framework that explains how and why a behavior occurs (e.g., learning theory, behavior change theory).
	Dialogue flow development	A psychotherapy approach (e.g., cognitive behavioral therapy, motivational interviewing) that serves as the basis for dialogue flow development. Can help enhance motivation for behavior change and identify and address barriers to physical activity.
	Intervention components	The elements and activities that make up the intervention program (e.g., health education, goal setting, workout planning, problem solving).
	Challenges and areas for improvement	Study limitations and areas in need of further research (e.g., ethical considerations, barriers to program development or implementation).
