# Supplementary material for: Promoting Physical Activity Through Conversational Agents: Mixed Methods Systematic Review
Source: J Med Internet Res. 2021 Sep 14;23(9):e25486. doi: 10.2196/25486 (PMC8479596; doi:10.2196/25486)
Supplement: Multimedia Appendix 4 [file jmir_v23i9e25486_app4.pdf]

Multimedia Appendix 4. Excluded studies with reasons for exclusion.

First author (year)	Title	Reason for exclusion
Ahtinen et al. (2010)	Let's all get up and walk to the North Pole: design and evaluation of a mobile wellness application	Wrong intervention (does not use a conversational agent)
Akker et al. (2011)	A self-learning personalized feedback agent for motivating physical activity	No outcome data
Albaina et al. (2009)	Flowie: a persuasive virtual coach to motivate elderly individuals to walk	Wrong intervention (does not use a conversational agent)
Bickmore (2003)	Relational agents: effecting change through human-computer relationships	Wrong article type (dissertation)
Bickmore et al. (2005)	Acceptance and usability of a relational agent interface by urban older adults	Not an original application
Bickmore et al. (2005)	Establishing and maintaining long-term human-computer relationships	Not an original application
Bickmore et al. (2008)	Negotiating task interruptions with virtual agents for health behavior change	Wrong patient problem (does not target physical activity)
Bickmore et al. (2009)	Context awareness in a handheld exercise agent	Wrong intervention (does not use a conversational agent)
Bickmore et al. (2010)	Usability of conversational agents by patients with inadequate health literacy: evidence from two clinical trials.	No outcome data
Biemans et al. (2010)	MyCoach: in situ user evaluation of a virtual and physical coach for running	Wrong intervention (does not use a conversational agent)
Clavel et al. (2018)	WEnner: a theoretically motivated approach for tailored coaching about physical activity	No outcome data
Eyck et al. (2006)	Effect of a virtual coach on athletes' motivation	Wrong intervention (does not use a conversational agent)
Fadhil et al. (2017)	An adaptive learning with gamification & conversational UIs: the rise of CiboPoliBot	Wrong patient problem (does not target physical activity)
Fasola et al. (2013)	A socially assistive robot exercise coach for the elderly	Wrong intervention (does not use a conversational agent)
Gardiner (2016)	Go to gabby for health and wellness: an embodied conversational agent addresses stress management information and coping techniques among diverse women in the inner city	No full text

Hahn et al. (2020)	Using virtual agents and activity monitors to autonomously track and assess self-determined physical activity among young children: a 6-week feasibility field study	Wrong intervention (does not use a conversational agent)
Heldt et al. (2018)	Telemedicine therapy for overweight adolescents: first results of a novel smartphone app intervention using a behavioural health platform	No full text
Hurling et al. (2009)	The benefits of (automated) dialogue	Not an original application
Kanaoka et al. (2015)	Designing a motivational agent for behavior change in physical activity	No full text
Kang et al. (2018)	"Give me the support I want!": the effect of matching an embodied conversational agent's social support to users' social support needs in fostering positive user-agent interaction	Wrong patient problem (does not target physical activity)
Klaassen et al. (2013)	Feedback presentation for mobile personalised digital physical activity coaching platforms	Wrong intervention (does not use a conversational agent)
Kramer et al. (2019)	Investigating intervention components and exploring states of receptivity for a smartphone app to promote physical activity: protocol of a microrandomized Trial.	No outcome data
Künzler et al. (2019)	Exploring the state-of-receptivity for mHealth interventions	Wrong outcomes
L'Allemand et al. (2018)	Design and interim evaluation of a smartphone app for overweight adolescents using a behavioural health intervention platform	No full text
Lane et al. (2015)	ZOE: a cloud-less dialog-enabled continuous sensing wearable exploiting heterogeneous computation	No outcome data
Lucas et al. (2018)	Effects of perceived agency and message tone in responding to a virtual personal trainer	Wrong intervention (does not use a conversational agent)
Ly et al. (2017)	A fully automated conversational agent for promoting mental well-being: a pilot RCT using mixed methods	Wrong patient problem (does not target physical activity)
Macek et al. (2010)	Pheasy - physical exercise assistance system - evaluation and usability study	Wrong outcomes
Makkar et al. (2018)	Conversational artificial intelligence for achieving activity targets through routine physical activity-longitudinal observational study among people with type 2 diabetes	No full text

Mavropoulos et al. (2019)	A smart dialogue-competent monitoring framework supporting people in rehabilitation	No outcome data
Murphy et al. (2020)	Delivery of a 3-month Mediterranean diet and physical activity lifestyle intervention via artificial-intelligence chatbot can achieve behaviour change: MedLiPal pilot-study.	Wrong article type
Pirolli et al. (2018)	Scaffolding the mastery of healthy behaviors with fittle+ systems: evidence-based interventions and theory	Not an original application
Pütten et al. (2011)	Living with a robot companion: empirical study on the interaction with an artificial health advisor	Wrong intervention (does not use a conversational agent)
Ren et al. (2014)	Supporting longitudinal change in many health behaviors	No full text
Saboo et al. (2019)	Wellthy care™, a digital therapeutic improves physical activity and logging patient journey data in patients with type-2 diabetes in India	No full text
Schneider et al. (2018)	Comparing the effects of social robots and virtual agents on exercising motivation	Wrong intervention (does not use a conversational agent)
Schulman et al. (2009)	Persuading users through counseling dialogue with a conversational agent	Wrong intervention (Wizard of Oz design)
Segerståhl et al. (2011)	Designing personal exercise monitoring employing multiple modes of delivery: implications from a qualitative study on heart rate monitoring.	Wrong intervention (does not use a conversational agent)
Sosale et al. (2018)	Real-world effectiveness of a digital therapeutic in improving glycaemic control in south Asians living with type 2 diabetes	No full text
Spitters et al. (2009)	Learning effective and engaging strategies for advice-giving human-machine dialogue	No outcome data
Stasinaki et al. (2018)	A novel digital health intervention improves physical performance in obese youth	No full text
Torres et al. (2019)	The EMPATHIC project: mid-term achievements	Wrong intervention (does not use a conversational agent)
Trinh et al. (2018)	Predicting user engagement in longitudinal interventions with virtual agents	Wrong outcomes
Tsiourti et al. (2014)	Virtual assistive companions for older adults: qualitative field study and design implications	Wrong patient problem (does not target physical activity)

Zia et al. (2019)	Personalized and unexpected rewards using a text-only artificial intelligence rewards platform can increase patient engagement and physical activity: TRUSTR pilot results underscore the need for intelligent patient-reported outcomes	No full text
