# Supplementary material for: Promoting Physical Activity Through Conversational Agents: Mixed Methods Systematic Review
Source: J Med Internet Res. 2021 Sep 14;23(9):e25486. doi: 10.2196/25486 (PMC8479596; doi:10.2196/25486)
Supplement: Multimedia Appendix 5 [file jmir_v23i9e25486_app5.pdf]

Multimedia Appendix 5. MMAT quality appraisal profile.

| | | | SCREENING | | 1. QUALITATIVE STUDIES | | | | |
|---|---|---|---|---|---|---|---|---|---|
| Citation | First author | Year | S1. Are there clear research questions or objectives? | S2. Do the collected data allow the research questions or objectives to be addressed? | 1.1. Is the qualitative approach appropriate to answer the research question? | 1.2. Are the qualitative data collection methods adequate to address the research question? | 1.3. Are the findings adequately derived from the data? | 1.4. Is the interpretation of results sufficiently substantiated by data? | 1.5. Is there coherence between qualitative data sources, collection, analysis and interpretation? |
| 12 | Bickmore | 2005 | Yes | Yes | Yes | Yes | Yes | Yes | Yes |
| 13 | Bickmore | 2005 | Yes | Yes | Yes | Can't tell | Yes | Yes | Yes |
| 14 | Bickmore | 2010 | Yes | Yes | | | | | |
| 30 | Bickmore | 2011 | Yes | Yes | Yes | Yes | No | No | No |
| 31 | Bickmore | 2013 | Yes | Yes | Yes | Yes | Yes | No | No |
| 32 | Bickmore | 2013 | Yes | Yes | | | | | |
| 33 | Fadhil | 2019 | Yes | Yes | | | | | |
| 34 | Fadhil | 2019 | Yes | Yes | | | | | |
| 35 | Friederichs | 2014 | Yes | Yes | | | | | |
| 36 | Gardiner | 2017 | Yes | Yes | Yes | Yes | Can't tell | Yes | Yes |
| 37 | Kocielnik | 2018 | Yes | Yes | Yes | Yes | Yes | Yes | Yes |
| 38 | Kramer | 2020 | Yes | Yes | | | | | |
| 39 | Maher | 2020 | Yes | Yes | | | | | |
| 40 | Olafsson | 2019 | Yes | Yes | Yes | Yes | Yes | Yes | Yes |
| 41 | Piao | 2020 | Yes | Yes | | | | | |
| 42 | Sillice | 2018 | Yes | Yes | Yes | Yes | Yes | Yes | Yes |
| 43 | Similä | 2014 | Yes | Yes | Yes | Yes | Can't tell | Can't tell | Can't tell |
| 44 | Vainio | 2014 | Yes | Yes | | | | | |
| 45 | Watson | 2012 | Yes | Yes | | | | | |
| 46 | Zhou | 2017 | Yes | Yes | Yes | Can't tell | Yes | Yes | Yes |

Multimedia Appendix 5. MMAT quality appraisal profile.

| Citation | First author | Year | 2. RANDOMIZED CONTROLLED TRIALS<br>2.1. Is randomization appropriately performed? | 2.2. Are the groups comparable at baseline? | 2.3. Are there complete outcome data? | 2.4. Are outcome assessors blinded to the intervention provided? | 2.5 Did the participants adhere to the assigned intervention? |
|---|---|---|---|---|---|---|---|
| 12 | Bickmore | 2005 | Can't tell | Yes | Yes | Can't tell | No |
| 13 | Bickmore | 2005 | Can't tell | Can't tell | Yes | Can't tell | Yes |
| 14 | Bickmore | 2010 | | | | | |
| 30 | Bickmore | 2011 | | | | | |
| 31 | Bickmore | 2013 | Can't tell | No | Yes | Can't tell | Yes |
| 32 | Bickmore | 2013 | Yes | Yes | Yes | Yes | Yes |
| 33 | Fadhil | 2019 | | | | | |
| 34 | Fadhil | 2019 | | | | | |
| 35 | Friederichs | 2014 | Can't tell | Yes | No | Can't tell | No |
| 36 | Gardiner | 2017 | Yes | Yes | Yes | Yes | Yes |
| 37 | Kocielnik | 2018 | | | | | |
| 38 | Kramer | 2020 | Can't tell | Can't tell | No | Can't tell | No |
| 39 | Maher | 2020 | | | | | |
| 40 | Olafsson | 2019 | | | | | |
| 41 | Piao | 2020 | Yes | Yes | Yes | Can't tell | Yes |
| 42 | Sillice | 2018 | | | | | |
| 43 | Similä | 2014 | | | | | |
| 44 | Vainio | 2014 | Can't tell | Yes | No | Can't tell | No |
| 45 | Watson | 2012 | Yes | Yes | Yes | No | Yes |
| 46 | Zhou | 2017 | | | | | |

Multimedia Appendix 5. MMAT quality appraisal profile.

| Citation | First author | Year | 3. NON-RANDOMIZED STUDIES | | | | |
|---|---|---|---|---|---|---|---|
| | | | 3.1. Are the participants representative of the target population? | 3.2. Are measurements appropriate regarding both the outcome and intervention (or exposure)? | 3.3. Are there complete outcome data? | 3.4. Are the confounders accounted for in the design and analysis? | 3.5. During the study period, is the intervention administered (or exposure occurred) as intended? |
| 12 | Bickmore | 2005 | | | | | |
| 13 | Bickmore | 2005 | | | | | |
| 14 | Bickmore | 2010 | Yes | Yes | Yes | Can't tell | Yes |
| 30 | Bickmore | 2011 | Can't tell | Yes | Yes | No | Yes |
| 31 | Bickmore | 2013 | | | | | |
| 32 | Bickmore | 2013 | | | | | |
| 33 | Fadhil | 2019 | Yes | Yes | Yes | Can't tell | Yes |
| 34 | Fadhil | 2019 | Yes | Yes | Yes | Can't tell | Yes |
| 35 | Friederichs | 2014 | | | | | |
| 36 | Gardiner | 2017 | | | | | |
| 37 | Kocielnik | 2018 | Can't tell | Yes | Yes | Can't tell | Yes |
| 38 | Kramer | 2020 | | | | | |
| 39 | Maher | 2020 | Yes | Yes | Yes | Can't tell | Yes |
| 40 | Olafsson | 2019 | No | Yes | Yes | Yes | Yes |
| 41 | Piao | 2020 | | | | | |
| 42 | Sillice | 2018 | | | | | |
| 43 | Similä | 2014 | | | | | |
| 44 | Vainio | 2014 | | | | | |
| 45 | Watson | 2012 | | | | | |
| 46 | Zhou | 2017 | Yes | Yes | Yes | Can't tell | Yes |

Multimedia Appendix 5. MMAT quality appraisal profile.

| Citation | First author | Year | 5. MIXED METHODS STUDIES<br>5.1. Is there an adequate rationale for using a mixed methods design to address the research question? | 5.2. Are the different components of the study effectively integrated to answer the research question? | 5.3. Are the outputs of the integration of qualitative and quantitative components adequately interpreted? | 5.4. Are divergences and inconsistencies between quantitative and qualitative results adequately addressed? | 5.5. Do the different components of the study adhere to the quality criteria of each tradition of the methods involved? | SCORE |
|---|---|---|---|---|---|---|---|---|
| 12 | Bickmore | 2005 | Yes | Yes | Yes | Yes | No | 40% |
| 13 | Bickmore | 2005 | No | Yes | Yes | Yes | No | 40% |
| 14 | Bickmore | 2010 | | | | | | 80% |
| 30 | Bickmore | 2011 | No | No | No | Can't tell | No | 0% |
| 31 | Bickmore | 2013 | Yes | Yes | Yes | No | No | 40% |
| 32 | Bickmore | 2013 | | | | | | 100% |
| 33 | Fadhil | 2019 | | | | | | 80% |
| 34 | Fadhil | 2019 | | | | | | 80% |
| 35 | Friederichs | 2014 | | | | | | 20% |
| 36 | Gardiner | 2017 | Yes | Yes | Yes | Yes | Yes | 80% |
| 37 | Kocielnik | 2018 | Yes | Yes | Yes | Can't tell | Yes | 60% |
| 38 | Kramer | 2020 | | | | | | 0% |
| 39 | Maher | 2020 | | | | | | 80% |
| 40 | Olafsson | 2019 | No | Yes | Yes | Can't tell | Yes | 60% |
| 41 | Piao | 2020 | | | | | | 80% |
| 42 | Sillice | 2018 | | | | | | 100% |
| 43 | Similä | 2014 | | | | | | 40% |
| 44 | Vainio | 2014 | | | | | | 20% |
| 45 | Watson | 2012 | | | | | | 80% |
| 46 | Zhou | 2017 | No | Yes | Yes | Can't tell | Yes | 60% |

Multimedia Appendix 5. MMAT quality appraisal profile.

## Appraisal Instructions

1) Enter information on the papers you are appraising.
   - •Citation: if you have several papers to appraise, adding a reference number can be useful.
   - •First author: family name of the first author.
   - •Year: year of publication of the paper.

2) Respond to the two screening questions.
   - •F and thus cannot be appraised using the MMAT.

3) Choose the appropriate categories of studies to appraise.
   - •For a qualitative study: choose the qualitative set of criteria (set #1).
   - •For a quantitative study: choose the appropriate quantitative set of criteria (either set #2, 3 or 4).
   - •F the appropriate quantitative set (either set #2, 3 or 4), and the mixed methods set (set #5).

4) Rate the criteria of the chosen category (or categories).
   - •'Yes' means that the criterion is met.
   - •'No' means that the criterion is not met.
   - •'Can't tell' means that there is not enough information in the paper for you to judge if the criterion is met or not.

## Scoring Instructions

For each retained study, an overall quality score may not be informative (in comparison to a descriptive summary using MMAT criteria), but might be calculated using the MMAT. Since there are only a few criteria for each domain, the score can be presented using descriptors such as stars (*) or %: 5***** or 100% quality criteria met; 4**** or 80% quality criteria met; 3*** or 60% quality criteria met; 2** or 40% quality criteria met; 1* or 20% quality criteria met.
For mixed methods studies, since there are 15 criteria to rate (instead of 5), the premise is that the overall quality of a combination cannot exceed the quality of its weakest component. Thus, the overall quality score is the lowest score of the study components. The score is 20% when QUAL=1, QUAN=1 or MM=1; it is 40% when QUAL=2, QUAN=2 or MM=2; it is 60% when QUAL=3, QUAN=3 or MM=3; it is 80% when QUAL=4, QUAN=4 or MM=4; and it is 100% when QUAL=5, QUAN=5, or MM=5.
