# Supplementary material for: Promoting Physical Activity Through Conversational Agents: Mixed Methods Systematic Review
Source: J Med Internet Res. 2021 Sep 14;23(9):e25486. doi: 10.2196/25486 (PMC8479596; doi:10.2196/25486)
Supplement: Multimedia Appendix 6 [file jmir_v23i9e25486_app6.pdf]

Multimedia Appendix 6. Characteristics of comparators in each included study.

Author (year)	Number of Groups	Intervention type(s)	Control type
Bickmore et al (2005)[12]	2	1) ECA	2) pedometer + printed material
Bickmore et al (2005)[13]	3	1) relational ECA; 2) non-relational ECA	3) web forms
Bickmore et al (2010)[14]	1	1) ECA	N/A
Bickmore et al (2011)[30]	1	1) ECA	N/A
Bickmore et al (2013)[31]	4	1) ECA targeting physical activity; 2) ECA targeting fruit/vegetable consumption; 3) ECA targeting both	4) pedometer
Bickmore et al (2013)[32]	2	1) ECA	2) pedometer
Fadhil & AbuRa'ed (2019)[33]	1	1) chatbot	N/A
Fadhil et al (2019)[34]	1	1) chatbot	N/A
Friederichs et al (2014)[35]	3	1) ECA; 2) chatbot	3) no intervention
Gardiner et al (2017)[36]	2	1) ECA	2) patient education worksheets + meditation CD/MP3
Kocielnick et al (2018)[37]	1	1) chatbot	N/A
Kramer et al (2020)[38]	3	1) chatbot + cash incentives; 2) chatbot + charity incentives	3) chatbot + no financial incentives
Maher et al (2020)[39]	1	1) chatbot	N/A
Olafsson et al (2019)[40]	4	1) ECA targeting physical activity with coerced change talk; 2) ECA targeting physical activity without coerced change talk; 3) ECA targeting diet with coerced change talk; 4) ECA targeting diet without coerced change talk	N/A
Piao et al (2020)[41]	2	1) chatbot + rewards	2) chatbot + no rewards
Sillice et al (2018)[42]	3	1) ECA + webpage; 2) webpage	3) no intervention

Simila et al (2014)[43]	1	1) ECA + chatbot	N/A
Vainio et al (2014)[44]	3	1) self-selected ECA; 2) fixed, neutral ECA	3) chatbot
Watson et al (2012)[45]	2	1) ECA	2) pedometer + website
Zhou et al (2017)[46]	2	1) American ECA; 2) Chinese ECA	N/A
